# Production of Herbicide-Sensitive Strain to Prevent Volunteer Rice Infestation Using a CRISPR-Cas9 Cytidine Deaminase Fusion

**DOI:** 10.3389/fpls.2020.00925

**Published:** 2020-08-05

**Authors:** Akira Komatsu, Miki Ohtake, Zenpei Shimatani, Keiji Nishida

**Affiliations:** ^1^ Institute of Agrobiological Sciences, National Agriculture and Food Research Organization (NARO), Tsukuba, Japan; ^2^ Graduate School of Science, Technology and Innovation, Kobe University, Kobe, Japan

**Keywords:** activation-induced cytidine deaminase (AID), *Oryza sativa* L., targeted nucleotide substitution, *HIS1*, volunteer rice, benzobicyclon, mesotrione, sulcotrione

## Abstract

When cultivated rice seed fall into fields, they may overwinter and spontaneously germinate the next spring. Such germinated plants are termed “volunteer rice.” Volunteer grains originating from feed rice varieties may differ in certain traits, such as quality and taste, as compared with those of rice cultivated for human consumption, which may reduce the overall quality of the final harvested grain. Many rice varieties show resistance to benzobicyclon (BBC), a beta-triketone herbicide (bTH) that inhibits 4-hydroxyphenylpyruvate dioxygenase (HPPD). Recently, the rice gene *HIS1* (*HPPD INHIBITOR SENSITIVE 1*) conferring resistance to BBC and other bTHs was identified. In this study, to suppress the occurrence of volunteer rice infestation, we attempted to generate a BBC-sensitive rice strain *via* the knockout of the *HIS1* gene using genome editing techniques. The production of a *his1* knockout line was carried out by the start-codon substitution or stop-codon creation using CRISPR-Cas9 cytidine deaminase fusion, which is useful as a novel amino acid sequence is not generated due to the shifting of the reading frame. The mutation frequencies of independent transgenic plants were 3.6, 13.5, 13.8, and 21.2% at four gRNAs for start-codon substitution and three stop-codon creations. The *his1* knockout lines were conferred with sensitivity to BBC, re-confirming by genome editing that this is indeed the gene responsible for BBC resistance/sensitivity. The *his1* knockout lines also exhibited a sensitive phenotype to other bTHs, including sulcotrione, mesotrione, tembotrione, and tefuryltrione, compared with the wild-type variety ‘Nipponbare.' These results demonstrate the potential of herbicide-sensitive rice produced by genome editing technology as a material to control volunteer feed rice using pre-labeled herbicides for varieties consumed by humans.

## Introduction

Genome editing technology has been attracting attention as a means to change and modify the target domains within genes/genomes in diverse species. Recently, the CRISPR-Cas9 system has been developed, which employs a Cas9 endonuclease and guide RNA complex, and has exhibited very high efficiency for target gene editing in various species (Cong et al., 2013; Feng et al., 2013; Jiang et al., 2013; Mali et al., 2013; Nekrasov et al., 2013; Shan et al., 2013). In addition, efforts are in progress to develop a novel genome editing technique that does not involve DNA double-strand breaks. Cytosine base editor (CBE) is one such technology, which employs activation-induced cytidine deaminase (AID) that catalyzes a deamination reaction and couples with molecules harboring DNA sequence recognition ability, thereby modifying the genome sequence *via* nucleotide substitution in domains containing specific DNA sequences. AID is an enzyme that converts cytosine within DNA/RNA into uracil through a deamination reaction. The resulting U-G DNA mismatches trigger nucleotide substitutions (C to T or G to A) through DNA replication and repair pathways. AID prefers single-strand DNA within RNA/DNA hybrid transcripts (R loops) as its substrate. By making complete use of nuclease-impaired CRISPR-Cas9 and AID-related deaminases, researchers have recently developed a BE using rat APOBEC1 (Komor et al., 2016) and Target-AID system using sea lamprey-derived PmCDA1 to demonstrate the direct installation of point mutations (Nishida et al., 2016). The mutation spectrum of Target-AID is highly specific; the system preferentially induces point mutations at cytosine bases within a 5-bp window surrounding the 18 bp upstream of the protospacer adjacent motif (PAM) sequence on the non-complementary strand to gRNA. The capability of Target-AID to avoid cutting genomic double-stranded DNA reduces off-target effects relating to CRISPR-Cas9 nuclease activity and the incidence of cellular cytotoxicity (Nishida et al., 2016). Target-AID system was also applied to higher plants to obtain desirable phenotypes in rice, tomatoes, wheat, and maize (Shimatani et al., 2017; Zong et al., 2017). Simultaneous editing of multiplex traits was also demonstrated in rice (Shimatani et al., 2018). Recently, rationally engineered Cas9 requiring NG-PAM sequences (Cas9-NG) was developed and shown to be compatible with Target-AID (Nishimasu et al., 2018), which was then applied to rice (Endo et al., 2019). Recently, adenine base editors (ABEs) were developed as a system for mediate the conversion of A to G or T to C in genomic DNA (Gaudelli et al., 2017). These systems, together with cytosine base editors by cytidine deaminase, enable introduction of transitions for four patterns (C to T, G to A, A to G, and T to C) at the target site in the genome, expanding the capabilities of base editing. In this manner, the base editing technology continues to evolve and is expected to be capable of achieving breeding goals more efficiently.

Rice is one of the most important crops in the world, with more than half of the global population relying upon it as a staple food (Sasaki, 2008). It is also essential as livestock feed. Worldwide, especially in advanced or emerging countries where the consumption of livestock meat is already high or rapidly increasing, domestic cultivation, and utilization of feed crops is key to maintaining self-sufficiency. Currently, a multitude of high-yield rice varieties have been cultivated specifically for livestock through agricultural breeding efforts, for instance, in Japan (Sakai et al., 2003; Kato, 2008). As the Japanese government encourages the cultivation and harvest of feed rice, farmers have formulated annual production plans by gauging the optimum ratio of edible rice (i.e., human consumption) to feed rice (i.e., livestock consumption) varieties. Under such circumstances, when seeds of cultivated rice migrate onto a paddy field, some of them may overwinter and germinate spontaneously on the following spring. Such germinated seedlings are called “volunteer rice” (Singh et al., 2017). The occurrence of volunteer rice originating from a feed rice variety results in the mixing of feed rice varieties within the edible rice crops to be grown the next year. Edible and feed varieties of rice considerably differ in terms of quality and taste. Hence, such a mixing may cause decline in the quality and lowering its sell prices of harvested rice grains intended for human consumption. To avoid quality loss by such a crop contamination, farmers have a tendency to intentionally cultivate low-yield edible rice varieties as feed rather than using higher-yield varieties bred specifically for livestock consumption.

Beta-triketone herbicides (bTHs) are 4-hydroxyphenylpyruvate dioxygenase (HPPD) inhibitors widely used in agriculture. Benzobicyclon (BBC) is a bTH developed for weed control in paddy fields and is effective against paddy weeds resistant to other types of herbicide, including sulfonylureas. BBC is a prodrug, with its hydrolysate, BBC-OH, is incorporated into plant, acting as an HPPD inhibitor, and mediating plant bleaching (Sekino et al., 2008). Recent studies have revealed that although many Japanese rice varieties show resistance to BBC, certain varieties, including some *Indica* cultivars, remain sensitive to BBC (Maeda et al., 2019). These findings contribute to the identification of rice genes that determine resistance and sensitivity relative to BBC. Specifically, the main quantitative trait locus (QTL) for BBC sensitivity situated on the second chromosome was identified through a QTL analysis of the BC_1_F_2_ population and chromosomes derived from BBC-sensitive and BBC-resistant rice varieties. As this QTL behaves as a single recessive trait, it was identified as a gene locus determining BBC sensitivity/resistance, with the corresponding wild-type gene being named *HIS1* (Maeda et al., 2019). In addition, an analysis of BBC-sensitive rice cultivar revealed that BBC-OH was absorbed into the roots and transferred to the foliar tissue, but it was not detected in the leaf tissues of the *HIS1*-transformed line, suggesting that HIS1 functions as an enzyme for BBC-OH degradation in plants (Maeda et al., 2019).

In this study, to preclude rice varieties intended for either human or livestock consumption from being mixed together due to field infestation by volunteer rice, we attempted to produce a BBC-sensitive *his1* rice line *via* the knockout of the *HIS1* gene through nucleotide substitution using a CRISPR-Cas9 cytidine deaminase fusion.

## Materials and Methods

### Plasmid Vector Construction

**Figure 1:**
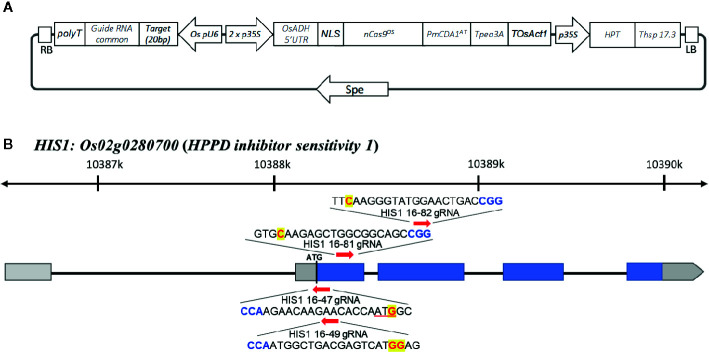
Target-AID vector and targeted sites of rice *HIS1* gene. **(A)** Schematic illustration for the Target-AID vector in this study. The engineered Cas9 nuclease *nCas9^Os^-PmCDA1^At^* is under the control of the doubled *cauliflower mosaic virus 35S* promoter, and transcription was terminated with the *Pea3A* terminator from *Pisum sativum*. The *U6* promoter of rice (*OsU6*) was used to drive the expression of gRNAs. **(B)** Schematic map of the gene structure of *HIS1* and the gRNA target sites. The blue and gray boxes indicate translated and untranslated regions (UTRs) on exon(s), respectively; arrows show the positions and direction of gRNA on *HIS1* gene for Target-AID. The PAM motif (NGG) is shown in blue. The target nucleotides for base substitution are shown in red.

The vectors used for the evaluation of herbicide resistance within rice were constructed as follows. First, a rice-optimized Target-AID vector (**Figure 1A**) was constructed. Rice-optimized *Streptococcus pyogenes* Cas9 (*SpCas9*) vector pZH_MMCas9 and gRNA construction vector pZK_OsU6-gRNA were kindly provided by Dr. Masaki Endo (Mikami et al., 2015), and a RuvC nuclease domain-deficient D10A mutation (nickase, *nCas9*) was introduced *via* PCR and Gibson Assembly methods (New England Biolabs, USA). A plant codon-optimized *PmCDA1* coding sequence was synthesized and inserted following *nCas9* using the same linker peptide as previously described (Nishida et al., 2016). This pZK_OsU6-sgRNA was modified to contain a *Mlu*I site after the *Spe*I site for ease of multiplexing. A target sgRNA sequence was inserted between the OsU6 promoter and sgRNA scaffold using PCR.

### Transformation and Regeneration

BBC-resistant *japonica* cultivar (*Oryza sativa* L cv. Nipponbare) was used in this study. The procedures for *Agrobacterium*-mediated transformation were performed as described previously (Toki, 1997; Toki et al., 2006). After co-cultivation of *Agrobacterium* carrying the Target-AID vector with rice scutellum-derived calli (pre-cultured for 5 days) for 3 days, infected calli were transferred to fresh callus induction medium (Toki et al., 2006) containing 50 mg/L hygromycin B (Wako Pure Chemicals) and 400 mg/L carbenicillin disodium salt (Nakarai, Kyoto, Japan) to remove residual *Agrobacterium*. At 21 days following hygromycin selection, proliferating calli were transferred to a fresh pre-regeneration medium containing 40 mg/L hygromycin and 200 mg/L carbenicillin disodium salt. After 8 days of culture, the calli were transferred to a fresh regeneration medium containing 30 mg/L hygromycin B and cultured for 2weeks. The regenerated plants were further cultivated in a greenhouse.

### Sequence Analysis

PCR products used for the restriction fragment length polymorphism analysis were also cloned into pCR-Blunt II-TOPO (Invitrogen) and subjected to a sequencing analysis using an ABI 3130 sequencer (Applied Biosystems).

### Herbicide Susceptibility Tests for Genome-Edited Lines *In Vitro*

The herbicide susceptibility of genome-edited lines was measured within test tubes (diameter, 2.5 cm; height, 15 cm) containing 10 ml of a Murashige–Skoog (MS) solid medium with herbicide. Five dehusked mature seeds of homozygous *his1* T_3_ or wild-type (control) rice plants were surface sterilized *via* two treatments with 4% sodium hypochlorite for 20 min followed by five rinses with sterilized water. The seeds were then immersed in sterilized water for 2 days at 30°C, after which germinated seeds were transferred to tubes containing the solid MS medium composed of half-strength MS salts and agar (1 g/L) containing herbicide; BBC 0.1 µM, BBC-OH 0.1 µM, and 0.3 µM; Mesotrione (MST); Sulcotrione (SLT); Tembotrione (TMT) 0.05 µM and 0.1 µM; and Tefuryltrione (TFT) 0.1 µM and 0.3 µM, cultured at 27°C for 7 to 14 days with 16 h of light (40 μmol m^−2^ s^−1^) daily. BBC and BBC-OH were obtained from SDS Biotech (Tokyo, Japan), while MST, SLT, TMT, and TFT were obtained from Fujifilm Wako (Tokyo, Japan).

### Evaluation of BBC and Other bTH Sensitivities of Genome-Edited Lines

The germination of Nipponbare (control) and genome-edited rice seeds was induced with incubation at 30°C for 2 to 3 days with 16-h light (40 μmol m^–2^ s^–1^) and 8-h dark photoperiods. Approximately 300 ml of soil specific for rice planting (Bonsol Baido, Sumitomo Kagaku, Tokyo, Japan) were mixed with water and deposited into plastic containers. Then, the germinated rice seeds were placed upon the soil surface. Seedlings were grown at 30°C for 7 to 10 days in a greenhouse, with the water level being maintained coincident with the soil surface. After the first leaves expanded, either water only or water containing herbicide (BBC: 0, 0.37, 0.75, 1.49, 2.24, and 4.48 µM; MST: 0.13 µM; SLT: 0.14 µM) was added to each cup so that the surface of the soil was 30 to 40 mm below that of the liquid. The liquid level was then maintained by occasional watering. Seedling growth was checked after 14 days.

## Results and Discussion

### Production of an *his1* Knockout Genome-Edited Line by Target-AID

**Figure 2:**
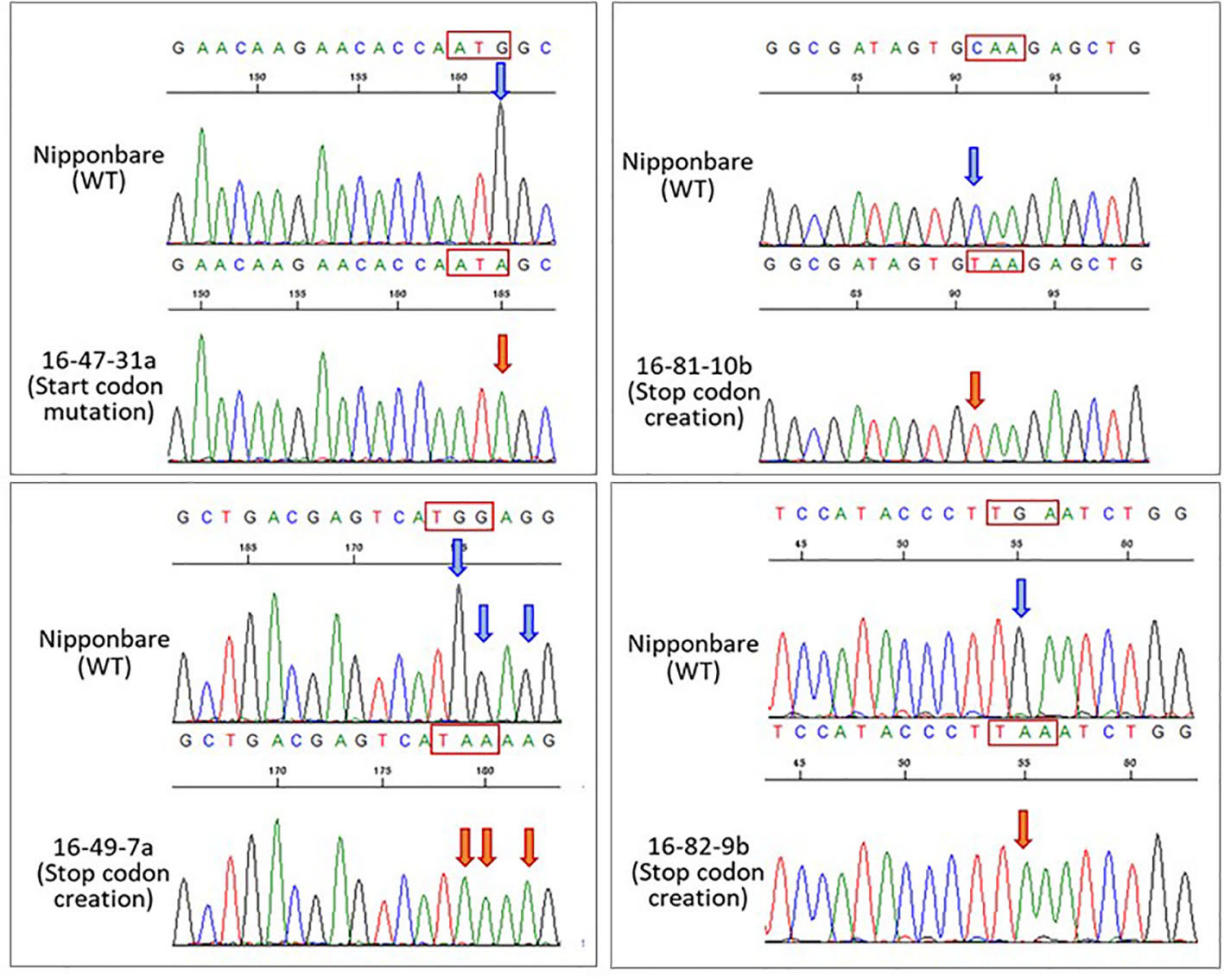
Sequencing chromatograms on nucleotide substitution regions in four *his1* lines. Sequencing chromatograms showing the nucleotide substitutions by Target-AID in 16-47, 16-49, 16-81, and 16-82 gRNAs in the *HIS1* gene of a representative T_2_ plant. Blue arrowheads indicate the wild-type nucleotide. Orange arrowheads indicate nucleotide substitutions. The nucleotide sequences in the red box indicate the target codon.

The Target-AID system was used to produce a *his1* knockout rice line (*his1* line). At four points on the *HIS1* gene, 20 bp gRNA sequences were designed (**Figure 1B**). One of such gRNA sequences (16-47 gRNA) introduced a mutation in the start-codon, with a G within the start-codon (ATG) on the second exon being substituted by A. Consequently, the starting methionine is converted to isoleucine (**Figure S1A**). This makes translation initiation impossible; therefore, we predicted that a knockout phenotype would emerge. The gRNA sequences at the three remaining points were designed using AID to create nonsense mutations: the first (16-49 gRNA) converts TGG-encoding tryptophan at the second exon into a stop-codon (TAA, TGA, or TAG; **Figure S1B**), the second (16-81 gRNA) is also situated on the second exon and converts CAA encoding a glutamine residue into a TAA stop-codon (**Figure S1C**), the final gRNA sequence (16-82 gRNA) is situated on the third exon and similarly converts CAA encoding a glutamine residue into a TAA stop-codon (**Figure S1D**). We used the Target-AID system carrying these gRNAs to edit the targets with the aim to facilitate the production of knockout plants. We investigated the efficiency of mutation with the 16-47 gRNA for the start-codon substitution and the other three gRNA sequences (16-49, 16-81, and 16-82 gRNAs) for the stop-codon creation. Following antibiotic hygromycin screening, 83, 52, 65, and 33 regenerated plants were obtained, respectively. For each mutation site, a sequence analysis was performed. Consequently, the mutation efficiency, including insertion, deletion, and substitution, was 12.0% for the 16-47 gRNA, 46.2% for the 16-49 gRNA, 36.9% for the 16-81 gRNA, and 36.4% for the 16-82 gRNA. In addition, the efficiency of indel integration was 9.6, 30.7, 7.7, and 9.1%, whereas the efficiency of the substitution alone was 3.6, 15.4, 29.2, and 27.3%. Finally, the number of lines achieving the targeted substitution rates was 3 (3.6%), 7 (13.5%), 9 (13.8%), and 7 (21.2%) (**Table 1**, **Figure 2**). Therefore, efficiency was the lowest for gRNA initiating the start-codon substitution. In addition, biallelic mutations with only base substitution were only found in one line (1.9%) with 16-49 gRNA.

**Table 1:** Mutation frequency at *HIS1* targets using Target-AID.

Variety	gRNA	Regenerated plants of Hyg^R^	Regenerated plants with indel mutants	Regenerated plants with substitution mutants	Regenerated plants with start codon mutation or creating stop codon
Nipponbare	*HIS1* 16-47 (Exon2)	83	8 (9.6%)	3 (3.6%)	3 (3.6%)
	*HIS1* 16-49 (Exon2)	52	16 (30.7%)	8 (15.4%)	7 (13.5%)
	*HIS1* 16-81 (Exon2)	65	5 (7.7%)	19 (29.2%)	9 (13.8%)
	*HIS1* 16-82 (Exon3)	33	3 (9.1%)	9 (27.3%)	7 (21.2%)

The transformed and hygromycin-resistant calli were analyzed by sequencing to identify mutation types. Percentage of T_0_ plants found with mutations in the target sequence.

As the efficiency of the start-codon substitution was lower than that of the stop-codon creation, in the latter case, as there are multiple sites for which gRNA can be designed within the exon domain, a more efficient substitution target site could be selected. However, for a start-codon substitution, only one predetermined site can be used to design gRNA, and it may not always be an optimal sequence.

The efficiency of the indel integration or point mutations using Target-AID in this study was similar to that previously reported for the *OsFTIP1e* gene (*nCas9^Os^-PmCDA1^At^*) in rice (Shimatani et al., 2017). In our previous report, we discovered that UGI suppresses indel formation and improves the targeted nucleotide substitution efficiency in mammalian cells (Nishida et al., 2016). However, no clear effect of UGI on indel frequency was confirmed in plant cells (Nishida and Shimatani, unpublished). Zhong et al. (2019) reported that the efficiencies of substitution mutations using Cas9-NG (D10A)-PmCDA1-UGI were 30 and 45%, respectively, which exceeds our maximum efficiency (29.2%). Indel frequency also tended to be slightly lower, suggesting the effect of UGI. Furthermore, a study by Tang et al. (2019) reported that the production efficiencies of base-substituted plants using STU-nCas-PmCDA1-UGI-tRNA were 38.9 and 68.8%, which are both higher than our reported production efficiency. However, the frequency of the indel lines were 16.7 and 25.0%, respectively, and some test plots were considerably higher than our results. These results suggest that the use of UGI does not necessarily lead to increased substitution efficiency by reducing indel frequency. Finally, the fact that multiple lines or strains containing start-codon substitutions or stop-codon creation mutations were produced for all four gRNA sequences demonstrates that the mutation efficiency is at practical levels in rice. In cases of start-codon substitutions or stop-codon creation mutations using Target-AID, no new amino acid sequence(s) caused by novel reading frames *via* frame shift are occurring. This case may be considered another advantage for approaches using Target-AID and other CBEs.

### Confirmation of Off-Target Mutations

The Target-AID system is used for nucleotide substitutions aimed at specific amino acid residue substitutions. As a result, there are often situations in which the use of gRNA with off-target candidates cannot be avoided. In this study, we investigated how many mismatches with gRNA sequences cause off-targeting.

A family of genes similar to the *HIS1* gene (*HIS1*-like gene: *HSL*) exists in the rice genome on the sixth chromosome in the form of five clusters. From the OsHSL proteins predicted in previous reports, Os06g0176700 (*HSL1A*) and Os06g0178700 (*HSL1B*) are the most similar to *HIS1* (87% sequence identity) (Maeda et al., 2019). We investigated the presence/absence of off-target mutations, including these genes. As a result, we confirmed an off-target mutation (11-base insertion) of *HSL1B* in one out of the three plants within the genome-edited line (16-47) targeting the start-codon sequence (**Table S1**). The 11-bp insertion site of *HSL1B* was predicted to be a region where cytidine deaminase would perform base substitution, and it was presumed that the 11 bp sequence before this region was inserted as a template. As a result, this region became a tandem repeat sequence due to the 11 bp insertion sequence (**Figure S2**).

Conversely, we observed no off-target mutations in the genome-edited lines for the creation of stop codons (**Tables S2–S4**). In the genome-edited plants generated with the 16-49 gRNA, no off-target mutations were observed despite the off-target candidate genes on the sixth chromosome having 100% homology with *HIS1*, except for the PAM sequences (**Table S2**). This result indicates that although the CRISPR-Cas9 system can target any genomic region, depending upon the gRNA design, the specificity of this system depends on the PAM located directly under the target sequence. These target sites must lie immediately 5′ of a PAM sequence that matches the canonical 5′-NGG form, although recognition at sites containing alternate PAM sequences (e.g., 5′-NAG) has also been reported, albeit at less efficient rates (Jinek et al., 2012; Jiang et al., 2013; Pattanayak et al., 2013).

As the *HIS1* gene targeted in this study exhibited a high level of homology with the *HSL* family, it seemed to be a case in which single-base substitution of *HIS1* is problematic. The data showed that only one plant had the off-target mutation (11-base insertion) on *HSL1B* in a start-codon substitution (gRNA: 16-47), with no other alternative codon. Thus, the creation of a base substitution in a specific codon sequence was found to infer more disadvantageous conditions for mutation efficiency and off-target mutations compared with obtaining indel mutant plants using CRISPR-Cas9. However, in this study, the acquisition of the target base substitution was demonstrated in all target regions and was without mutation(s) for any candidate genes at the off-target sites. Even if off-target mutations occur rarely, they can be detected and eliminated during the selection process in crop breeding. This finding indicates that the base substitution within the target region of the rice genome has been extremely difficult for conventional breeding, but it has been made possible using Target-AID. This development can lead to more efficient and precise breeding in the future.

### Evaluation of BBC and Other β-Triketone Sensitivities *In Vitro*

**Figure 3:**
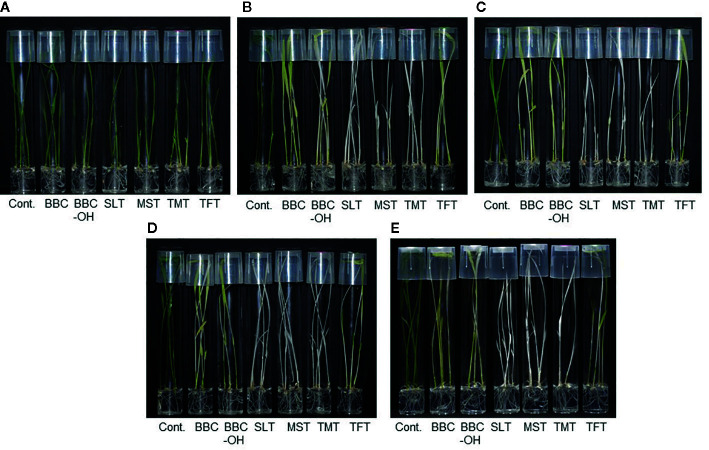
Herbicide susceptibility of *HIS1* genome-edited rice lines. Wild-type Nipponbare **(A)**, *his1* knockout lines by nucleotide substitution, *HIS1-*16-47-31a **(B)**, *HIS1*-16-49-7a **(C)**, *HIS1*-16-81-10b **(D)**, and *HIS1*-16-82-9b **(E)**. Homozygous T_3_ rice seeds were germinated and grown on Murashige–Skoog (MS) solid medium in the absence or presence of BBC (0.3 μM), BBC-OH (0.3 μM), MST (0.1 μM), SLT (0.1 μM), TMT (0.1 μM), or TFT (0.3 μM).

We created knockout rice lines by introducing mutations into *HIS1 via* base editing followed by the evaluation of their BBC sensitivity. For these analyses, the BBC-resistant cultivar ‘Nipponbare' was used as an original variety and homozygous T_3_ plants were used for the genome-edited line. The 16-47-31a (single-base substitution) and 16-47-32b (12 base deletions) lines carried mutations in the start-codon “ATG” composed of the 16-47 gRNA among the four gRNA sequences designed for *HIS1* (**Table 2**). In contrast to the wild-type ‘Nipponbare' exhibiting BBC resistance, these lines apparently acquired sensitivity to 0.1 μM BBC and its hydrolysate, BBC-OH within a concentration ranging from 0.1 to 0.3 μM (**Table 3**, **Figures 3A, B**). Meanwhile, the 16-47-20b line with a seven-base insertion mutation before the start-codon did not exhibit BBC sensitivity (**Table 3**).

**Table 2:** Mutation pattern of genome-edited lines used for evaluation of BBC and other β-triketone sensitivity.

gRNA	*HIS1* genome-edited line	Mutation pattern	
16-47 gRNA	Wild type	GAT**CCA**AGAACAAGAACACCA**ATG**G	
	16-47-20b	GAT**CCA**AGAACAAGAACACC**GAACACC**AATGG	7-base insertion (before ATG)
	16-47-31a	GAT**CCA**AGAACAAGAACACCAAT**A**G	1-base substitution (ATG → ATA)
	16-47-32b	GAT**CCA**AGAACAA– – – – – – – – – – – – CTG	12-base deletion (including ATG)
16-49 gRNA	Wild type	AACA**CCAATG**GCTGACGAGTCAT**GG**AGGG	
	16-49-1a	AACA**CC** – – – – – – – – – – – – – – – – – – – – – GG	21-base deletion (including ATG)
	16-49-7a	AACA**CCAATG**GCTGACGAGTCAT**AA**A**A**GG	3-base substitution (stop codon creation)
	16-49-14b	AACA**CCAATG**GC – – – – – – – – – – – – – – – – – – – – CGG	12-base deletion (including ATG)
	16-49-28a	AACA**CCAATG**GCTGACGAGTCAT**CA**A**A**GG	3-base substitution (Trp Arg → Ser Lys)
16-81 gRNA	Wild type	GGCGATAGTG**C**AAGAGCTGGCGGCAGC**CGG**	
	16-81-4b	GGCGATAGTG**G**AAGAGCTGGCGGCAGC**CGG**	1-base substitution (Gln → Glu)
	16-81-9b	GGCGATAGTGCAAGAGC – – – – – – –AGC**CGG**	7-base deletion
	16-81-10b	GGCGATAGTG**T**AAGAGCTGGCGGCAGC**CGG**	1-base substitution (stop codon creation)
16-82 gRNA	Wild type	TTCCAGATT**C**AAGGGTATGGAACTGAC**CGG**	
	16-82-4b	TTCCAGATT**G**AAGGGTATGGAACTGAC**CGG**	1-base substitution (Gln → Glu)
	16-82-9b	TTCCAGATT**T**AAGGGTATGGAACTGAC**CGG**	1-base substitution (stop codon creation)
	16-82-9c	TTCCAGATTCAAGG – – – – – – – – – – –AC**CGG**	11-base deletion

The target nucleotide for base substitution is shown in red. The PAM motif (NGG) is shown in a light blue highlight. Mutation of nucleotide substitution is shown in a green highlight. Mutation by insertion is shown in blue. Mutations by deletion are shown in ‐‐‐‐. The yellow highlights show the start-codon.

**Table 3:** Evaluation of BBC and other β-triketone sensitivity *in vitro*.

gRNA	*HIS1* genome-edited line	Mutation pattern	BBC	BBC-OH		Mesotrione		Sulcotrione		Tembotrione		Tefuryltrione	
			0.1μM	0.1μM	0.3μM	0.05μM 0.1μM		0.05μM 0.1μM		0.05μM 0.1μM		0.1μM 0.3μM	

16-47 gRNA	16-47-20b	7-base insertion (before ATG)	R	R	R	R	R	RS	R	RS	RS	RS	R
	16-47-31a	1-base substitution (ATG → ATA)	S	S	S	SS	SS	S	SS	S	SS	S	S
	16-47-32b	12-base deletion (including ATG)	S	S	S	SS	SS	S	SS	S	SS	S	S
16-49 gRNA	16-49-1a	21-base deletion (including ATG)	S	S	S	SS	SS	S	SS	S	SS	S	S
	16-49-7a	3-base substitution (stop codon creation)	S	S	S	SS	SS	S	SS	S	SS	S	S
	16-49-14b	12-base deletion (including ATG)	S	S	S	SS	SS	S	SS	S	SS	S	S
	16-49-28a	3-base substitution (Trp Arg → Ser Lys)	RS	RS	RS	RS	S	RS	S	RS	S	RS	RS
16-81 gRNA	16-81-4b	1-base substitution (Gln → Glu)	S	RS	S	R	R	RS	S	RS	S	RS	S
	16-81-9b	7-base deletion	S	S	S	SS	SS	S	SS	S	SS	S	S
	16-81-10b	1-base substitution (stop codon creation)	S	S	S	SS	SS	S	SS	S	SS	S	S
16-82 gRNA	16-82-4b	1-base substitution (Gln → Glu)	R	R	R	R	R	R	R	R	R	R	R
	16-82-9b	1-base substitution (stop codon creation)	S	S	S	SS	SS	S	SS	S	SS	S	S
	16-82-9c	11-base deletion	S	S	S	SS	SS	S	SS	S	SS	S	S
Nipponbare		Wild type	R	R	R	R	R	R	R	R	R	R	R

Herbicide susceptibility of genome editing lines was tested in test tubes. SS: Leaves completely whitened (Sensitivity). S: Whitening, light green (Sensitivity). RS: Green (Middle of sensitivity and resistance). R: Dark green (Resistance).

The base substitution lines generated by Target-AID; 16-49-7a, 16-81-10b, and 16-82-9b were made to create a stop-codon on the second or third exon. In a 0.1-µM BBC and a BBC-OH concentration ranging from 0.1 to 0.3 μM, these lines were markedly more sensitive as compared with the wild-type (**Table 3**, **Figures 3C–E**). Similarly, the deletion line on the second or third exon; 16-49-1a, 16-49-14b, 16-81-9b, and 16-82-9c also displayed BBC and BBC-OH sensitivity. There was no difference in susceptibility to BBC and its hydroxide BBC-OH among sensitive strains edited by genome editing. This supports the results of Sekino et al. (2008) and Maeda et al. (2019), in which BBC is a prodrug and the BBC-OH, is incorporated into the plant and functions as an HPPD inhibitor, mediating plant bleaching.

**Figure 4:**
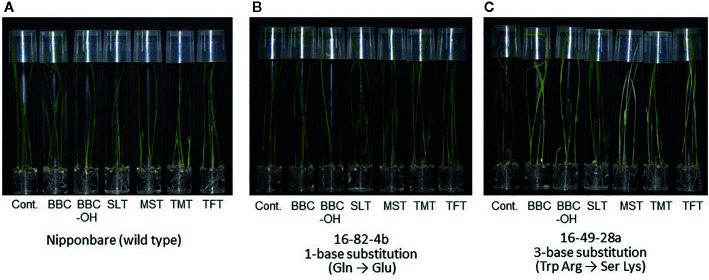
Herbicide susceptibility of *HIS1* genome-edited rice lines. Herbicide susceptibility of wild-type Nipponbare **(A)**, single-base (1 aa) substitution line (16-82-4b) using Nipponbare with *HIS1-*16-82 as gRNA **(B)**, and three-base (2 aa) substitution line (16-49-28a) using Nipponbare with *HIS1-*16-49 as gRNA **(C)**. Homozygous T_3_ rice seeds were germinated and grown on MS solid medium in the absence or presence of BBC (0.3 μM), BBC-OH (0.3 μM), MST (0.1 μM), SLT (0.1 μM), TMT (0.1 μM), or TFT (0.3 μM).

By contrast, in the 16-49-28a line using the 16-49 gRNA and the 16-82-4b line using the 16-82 gRNA, a stop-codon was not created because a substitution mutation to different nucleotide from the purpose in the target region (C to G substitution). Therefore, their resistance to BBC was maintained (**Table 3**, **Figure 4**). Previous studies have shown that C to G substitutions using Target-AID occurred relatively frequently behind to C to T substitutions in yeast (Nishida et al., 2016). In addition, C to G substitution was also observed in the *OsFTIP1e* gene of rice (Shimatani et al., 2018). The use of UGI may improve efficiency while restricting C to T nucleotide substitution (Komor et al., 2016; Nishida et al., 2016; Endo et al., 2019). We are also currently examining the effect of UGI on nucleotide substitution in rice.

All lines sensitive to BBC were also sensitive to bTHs, including mesotrione (MST), sulcotrione (SLT), tembotrione (TMT), and tefuryltrione (TFT) (**Table 3**, **Figure 3**). The lines that underwent a base substitution to a different nucleotide from the target retained resistance to bTHs. Unlike other bTHs, BBC-sensitive lines were sensitive to higher concentration(s) of TFT. A previous study showed that transformed lines expressing *HSL1* did not manifest BBC resistance, however they showed increased resistance to TFT (Maeda et al., 2019). The mechanism of action of TFT may be slightly different from other bTHs.

These findings reconfirmed the previously reported result that *HIS1* is the primary gene determining BBC sensitivity/resistance while using knockout lines created by genome editing (Maeda et al., 2019). In addition, these results thus suggested the possible application of *his1* and genome editing to the breeding of crops sensitive to multiple bTHs.

### Evaluation of BBC and Other β-Triketone Sensitivity in a Greenhouse

**Figure 5:**
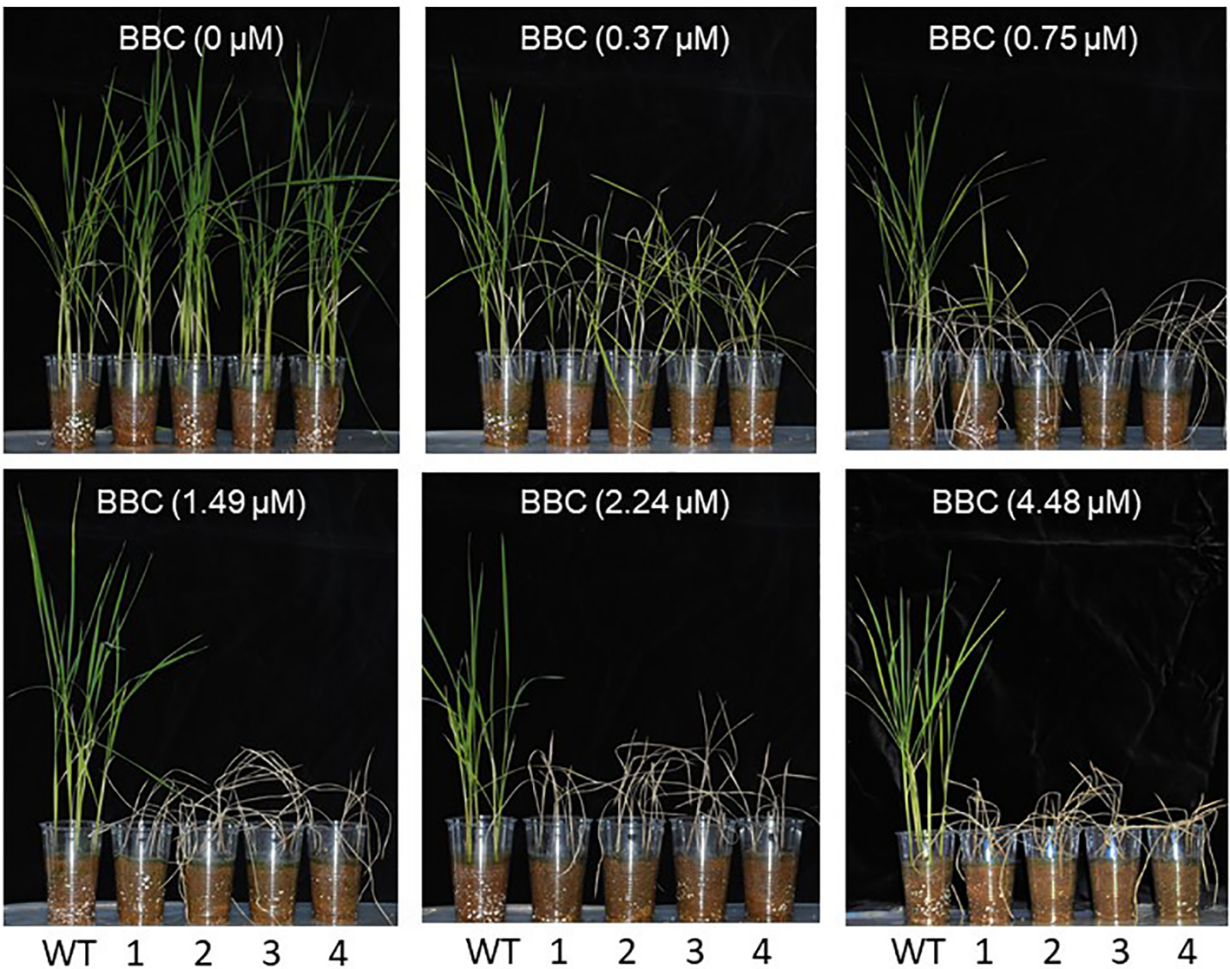
Concentration-dependent effects of BBC on the cultivar Nipponbare (WT, *HIS1*) and *his1* homozygous mutant lines (T_3_). 16-47-31a (1; start-codon mutant), 16-49-7a (2; stop-codon creation), 16-81-10b (3; stop-codon creation), and 16-82-9b (4; stop-codon creation) were grown in a controlled environment.

**Figure 6:**
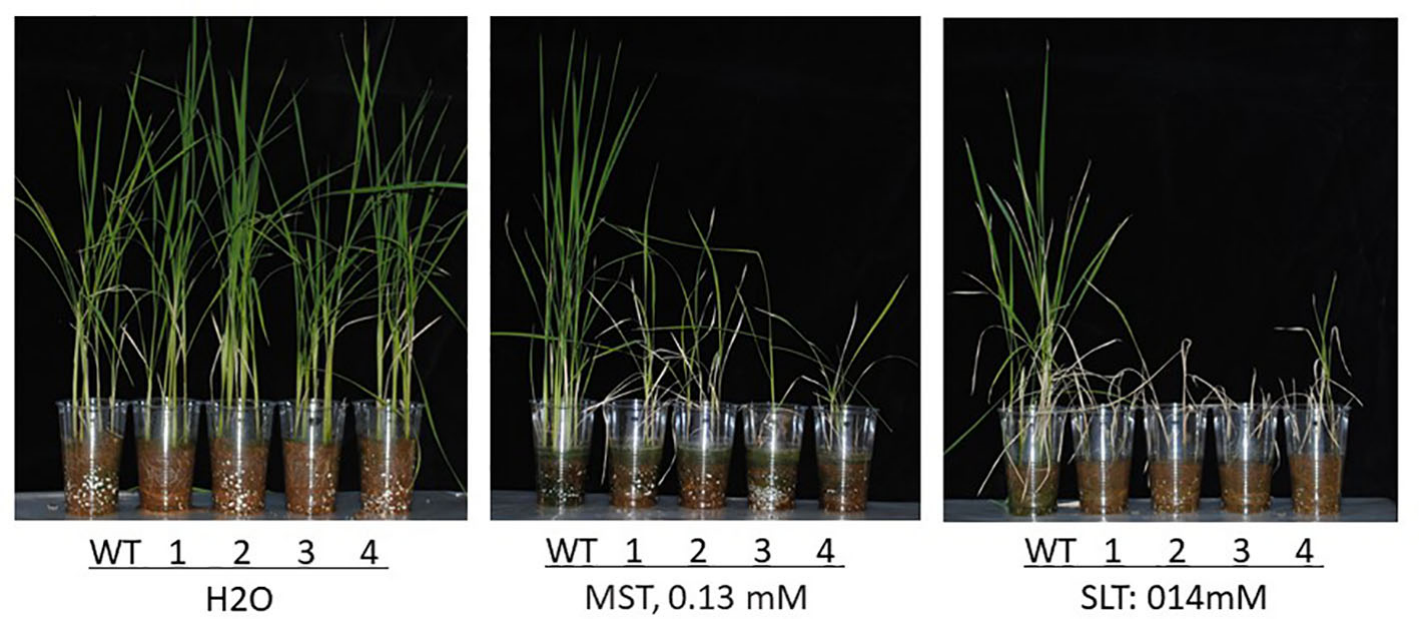
Effects of bTHs (MST, 0.13 mM; SLT, 0.14 mM) on Nipponbare (WT, *HIS1*) and the *his1* homozygous mutant lines (T_3_). 16-47-31a (1; start-codon mutant), 16-49-7a (2; stop-codon creation), 16-81-10b (3; stop-codon creation), and 16-82-9b (4; stop-codon creation) were grown in a controlled environment.

In a temperature-controlled greenhouse, we evaluated sensitivity to BBC and other β-triketones using the base-edited lines, including a T_3_ *his*1 homozygous line with a start-codon substitution and three lines with a stop-codon creation. Consequently, as with *in vitro* testing, we confirmed withering due to BBC sensitivity in the *his1* base substitution line with varying BBC concentrations (**Figure 5**). Meanwhile, in a test pot at 0.37 μM BBC concentration, no deaths were observed, although growth was suppressed. These base substitution lines also exhibited sensitivity to other bTHs, MST (0.13µM), and SLT (0.14 µM) (**Figure 6**). This finding indicates that genome editing can produce *his1* knockout rice varieties in a short period of time, including varieties and strains that acquired sensitivity to BBC and other bTHs.

**Figure 7:**
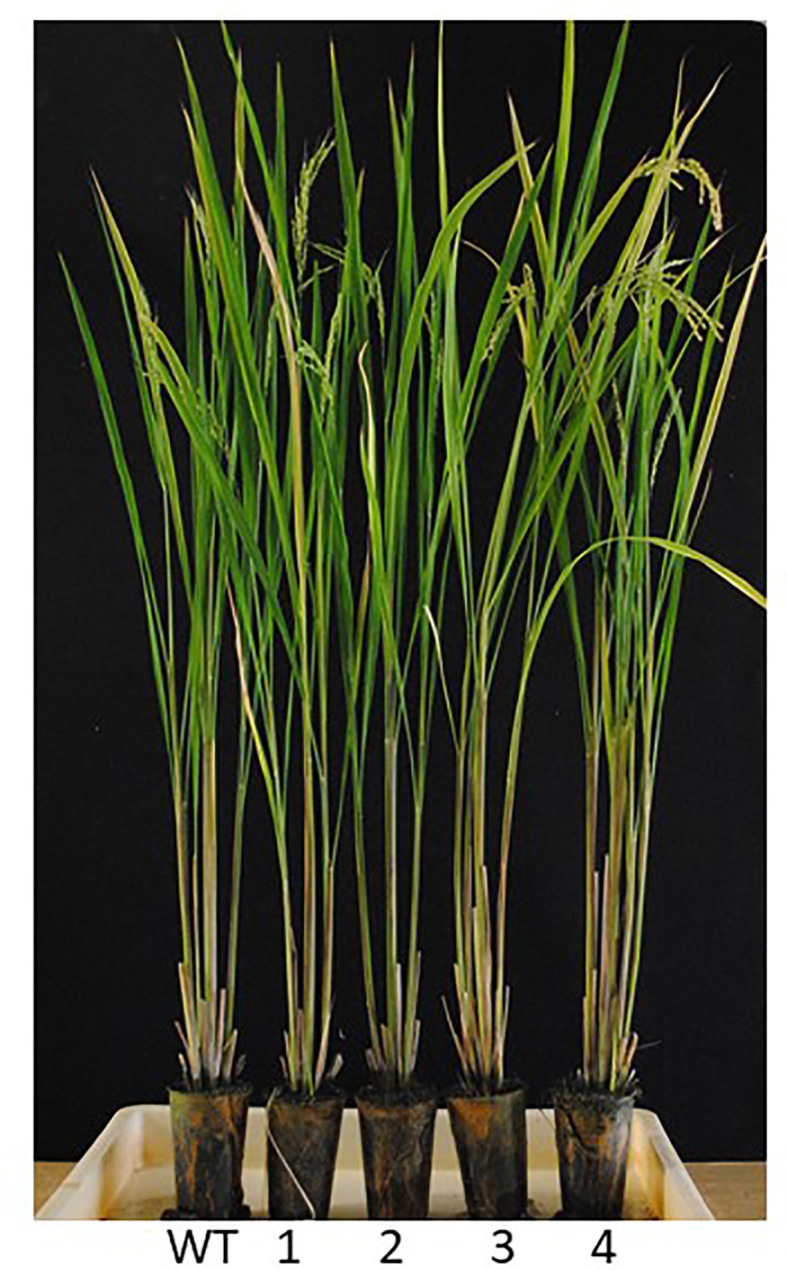
T_3_ plants of homozygous *his1* lines. Nipponbare (WT, *HIS1*), the *his1* homozygous mutant lines (T_3_) 16-47-31a (1; start-codon mutation), 16-49-7a (2; stop-codon creation), 16-81-10b (3; stop-codon creation), and 16-82-9b (4; stop-codon creation) were grown in a controlled environment.

These results indicate that varieties and strains having acquired sensitivity to BBC and other bTHs can be produced quickly by *his1* knockout using genome editing techniques, including Target-AID. We also performed a sensitivity evaluation using commercially available herbicides containing either BBC or MST. Consequently, plant death was observed in all *HIS1* genome-edited lines used in the experiment (data not shown). In addition, phenotypes within the temperature-controlled greenhouse for base substitution lines used for evaluation of BBC and other β-triketone sensitivities were not abnormal compared with the wild-type (**Figure 7**). This desirable result suggests that the *his1* rice line is not drastically affected by knockout of *HIS1* (e.g., the pleiotropic effect of reduced expression). Conversely, the original role of his1 in rice plants has not been elucidated yet. In the future, a more robust assessment of the agronomic impacts of pinpoint his1 knockouts versus WT lines in the same genome background under field conditions should be conducted. Such *his1* lines can quickly respond to the agricultural goal of suppressing volunteer rice field infestation. Given these results, a further evaluation of BBC sensitivity by cultivation in outdoor paddy fields should be conducted.

In this report, *his1* knockout lines were created using base-editing upon the BBC-resistant variety ‘Nipponbare' with almost all lines acquiring BBC sensitivity. At present, we are attempting to produce additional *his1* knockout lines *via* Target-AID using a BBC-resistant practical variety specifically for livestock feed (data not shown). One of the strategic advantages of suppressing the occurrence of volunteer rice by using BBC and other bTHs is that most *japonica* rice varieties grown for human consumption are resistant to these herbicides. In addition, pinpoint gene editing will be advantageous because it can avoid the linkage of vicinity agronomically problematic traits (linkage drags) caused by conventional crossbreeding. In the future, the production of feed rice varieties with the control of volunteer rice infestation will lead to further cultivation and utilization of high-yield feed rice varieties.

## Conclusion

To reduce the occurrence of volunteer rice field infestation, we created rice strains sensitive to the herbicide BBC *via* genome editing. A strategy for start-codon substitution and stop-codon creation by the targeted-AID system was used to create a *his1* knockout lines. As a result, *his1* knockout lines with nucleotide substitution showed sensitivity to BBC and other β-triketones. These strains are expected to contribute to the suppression of volunteer rice following spraying of BBC and other β-triketones.

## Author's Note

All authors declare that this study adheres to standard biosecurity and institutional safety procedures.

## Data Availability Statement

All datasets presented in this study are included in the article/**Supplementary Material**.

## Author Contributions

AK and KN designed the study, and KN and ZS designed gRNAs and constructed the Target-AID vector. AK and MO carried out the transgenic plant generation, DNA sequence analysis, and evaluation of the herbicide-sensitive strains. AK and KN wrote the manuscript. All authors contributed to the article and approved the submitted version.

## Funding

This research was supported by a grant from the Bio-oriented Technology Research Advancement Institution, NARO (Research Program on Development of Innovative Technology, 29009B).

## Conflict of Interest

The authors declare that the research was conducted in the absence of any commercial or financial relationships that could be construed as a potential conflict of interest.

## References

[B1] Cong L. Ran F. A. Cox D. Lin S. Barretto R. Habib N. (2013). Multiplex genome engineering using CRISPR/Cas systems. Science 339, 819–823. 10.1126/science.1231143 23287718 PMC3795411

[B2] Endo M. Mikami M. Endo A. Kaya H. Itoh T. Nishimasu H. (2019). Genome editing in plants by engineered CRISPR-Cas9 recognizing NG PAM. Nat. Plants 5 (1), 14–17. 10.1038/s41477-018-0321-8 30531939

[B3] Feng Z. Zhang B. Ding W. Liu X. Yang D. L. Wei P. (2013). Efficient genome editing in plants using a CRISPR/Cas system. Cell Res. 23, 1229–1232. 10.1038/cr.2013.114 23958582 PMC3790235

[B4] Gaudelli N. M. Komor A. C. Rees H. A. Packer M. S. Badran A. H. Bryson D. I. (2017). Programmable base editing of A•T to G•C in genomic DNA without DNA cleavage. Nature 551, 464–471. 10.1038/nature24644 29160308 PMC5726555

[B5] Jiang W. Bikard D. Cox D. Zhang F. Marraffini L. A. (2013). RNA-guided editing of bacterial genomes using CRISPR-Cas systems. Nat. Biotechnol. 31, 233–239. 10.1038/nbt.2508 23360965 PMC3748948

[B6] Jinek M. Chylinski K. Fonfara I. Hauer M. Doudna J. A. Charpentier E. (2012). RNA-guided DNA endonuclease in adaptive bacterial immunity. Science 337, 816–821. 10.1126/science.1225829 22745249 PMC6286148

[B7] Kato H. (2008). Development of Rice Varieties for Whole Crop Silage (WCS) in Japan.

[B8] Komor A. C. Kim Y. B. Packer M. S. Zuris J. A. Liu D. R. (2016). Programmable editing of a target base in genomic DNA without double-stranded DNA cleavage. Nature 533, 420–424. 10.1038/nature17946 27096365 PMC4873371

[B9] Maeda H. Murata K. Sakuma N. Takei S. Yamazaki A. Karim M. R. (2019). A rice gene that confers broad-spectrum resistance to β-triketone herbicides. Science 365, 393–396. 10.1126/science.aax0379 31346065

[B10] Mali P. Yang L. Esvelt K. M. Aach J. Guell M. DiCarlo J. E. (2013). RNA-guided human genome engineering via Cas9. Science 339, 823–826. 10.1126/science.1232033 23287722 PMC3712628

[B11] Mikami M. Toki S. Endo M. (2015). Comparison of CRISPR/Cas9 expression constructs for efficient targeted mutagenesis in rice. Plant Mol. Biol. 88, 561–572. 10.1007/s11103-015-0342-x 26188471 PMC4523696

[B12] Nekrasov V. Staskawicz B. Weigel D. Jones J. D. Kamoun S. (2013). Targeted mutagenesis in the model plant Nicotiana benthamiana using Cas9 RNA-guided endonuclease. Nat. Biotechnol. 31, 691–693. 10.1038/nbt.2655 23929340

[B13] Nishida K. Arazoe T. Yachie N. Banno S. Kakimoto M. Tabata M. (2016). Targeted nucleotide editing using hybrid prokaryotic and vertebrate adaptive immune systems. Science 353, aaf8729. 10.1126/science.aaf8729 27492474

[B14] Nishimasu H. Shi X. Ishiguro S. Gao L. Hirano S. Okazaki S. (2018). Engineered CRISPR-Cas9 nuclease with expanded targeting space. Science 361, 1259–1262. 10.1126/science.aas9129 30166441 PMC6368452

[B15] Pattanayak V. Lin S. Guilinger J. P. Ma E. Doudna J. A. Liu D. R. (2013). High-throughput profiling of off-target DNA cleavage reveals RNA-programmed Cas9 nuclease specificity. Nat. Biotechnol. 31, 839–843. 10.1038/nbt.2673 23934178 PMC3782611

[B16] Sakai M. Iida S. Maeda H. Sunohara Y. Nemoto H. Imbe T. (2003). New rice varieties for whole crop silage use in Japan. Breed. Sci. 53, 271–275. 10.1270/jsbbs.53.271

[B17] Sasaki T. (2008). From the editor's desk. Rice 1, 1–2. 10.1007/s12284-008-9010-y

[B18] Sekino K. Koyanagi H. Ikuta E. Yamada Y. (2008). Herbicidal activity of a new paddy bleaching herbicide, benzobicyclon. J. Pestic. Sci. 33, 364–370. 10.1584/jpestics.G08-11

[B19] Shan Q. Wang Y. Li J. Zhang Y. Chen K. Liang Z. (2013). Targeted genome modification of crop plants using a CRISPR-Cas system. Nat. Biotechnol. 31, 686–688. 10.1038/nbt.2650 23929338

[B20] Shimatani Z. Kashojiya S. Takayama M. Terada R. Arazoe T. Ishii H. (2017). Targeted base editing in rice and tomato using a CRISPR-Cas9 cytidine deaminasefusion. Nat. Biotechnol. 35, 441–443. 10.1038/nbt.3833 28346401

[B21] Shimatani Z. Fujikura U. Ishii H. Matsui Y. Suzuki M. Ueke Y. (2018). Inheritance of co-edited genes by CRISPR-based targeted nucleotide substitutions in rice. Plant Physiol. Biochem. 131, 78–83. 10.1016/j.plaphy.2018.04.028 29778643

[B22] Singh V. Burgos N. R. Singh S. Gealy D. R. Gbur E. E. Caicedo A. L. (2017). Impact of volunteer rice infestation on yield and grain quality of rice. Pest Manag. Sci. 73 (3), 604–615. 10.1002/ps.4343 27328627

[B23] Tang X. Ren Q. Yang L. Bao Y. Zhong Z. He Y. (2019). Single transcript unit CRISPR 2.0 systems for robust Cas9 and Cas12a mediated plant genome editing. Plant Biotechnol. J. 7, 1431–1445. 10.1111/pbi.13068 PMC6576101 30582653

[B24] Toki S. Hara N. Ono K. Onodera H. Tagiri A. Oka S. (2006). Early infection of scutellum tissue with Agrobacterium allows high-speed transformation of rice. Plant J. 47, 969–976. 10.1111/j.1365-313X.2006.02836.x 16961734

[B25] Toki S. (1997). Rapid and efficient Agrobacterium-mediated transformation in rice. Plant Mol. Biol. Rep. 15, 16–21. 10.1007/BF02772109

[B26] Zhong Z. Sretenovic S. Ren Q. Yang L. Bao Y. Qi C. (2019). Improving plant genome editing with high-fidelity xCas9 and non-canonical PAM-targeting Cas9-NG. Mol. Plant 12, 1027–1036. 10.1016/j.molp.2019.03.011 30928637

[B27] Zong Y. Wang Y. Li C. Zhang R. Chen K. Ran Y. (2017). Precise base editing in rice, wheat and maize with a Cas9-cytidine deaminase fusion. Nat. Biotechnol. 35, 438–440. 10.1038/nbt.3811 28244994

