# Supplementary figures and images for: Production of Herbicide-Sensitive Strain to Prevent Volunteer Rice Infestation Using a CRISPR-Cas9 Cytidine Deaminase Fusion
Source: Front Plant Sci. 2020 Aug 5;11:925. doi: 10.3389/fpls.2020.00925 (PMC7419996; doi:10.3389/fpls.2020.00925)

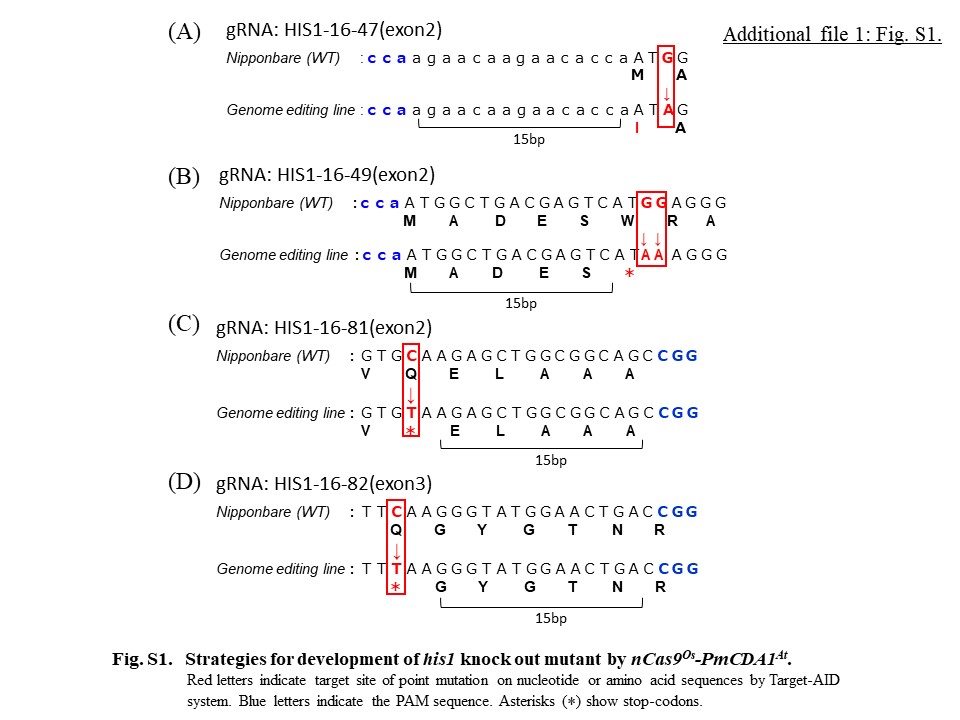

Supplement: Supplementary file 1 [file DataSheet_1.zip › Fig. S1..JPG]

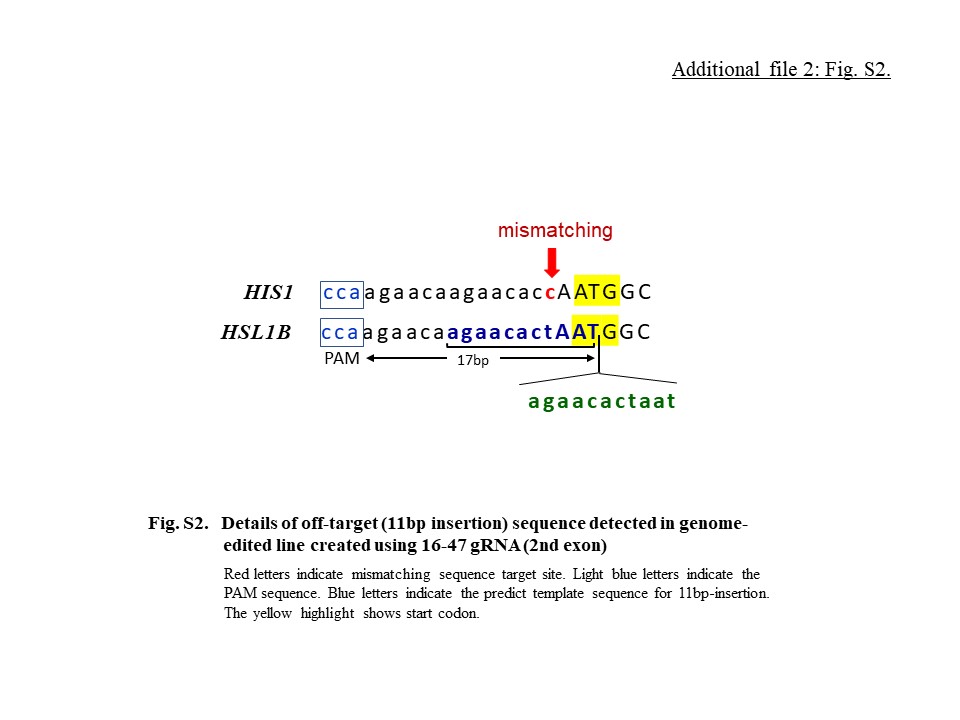

Supplement: Supplementary file 1 [file DataSheet_1.zip › Fig. S2..JPG]

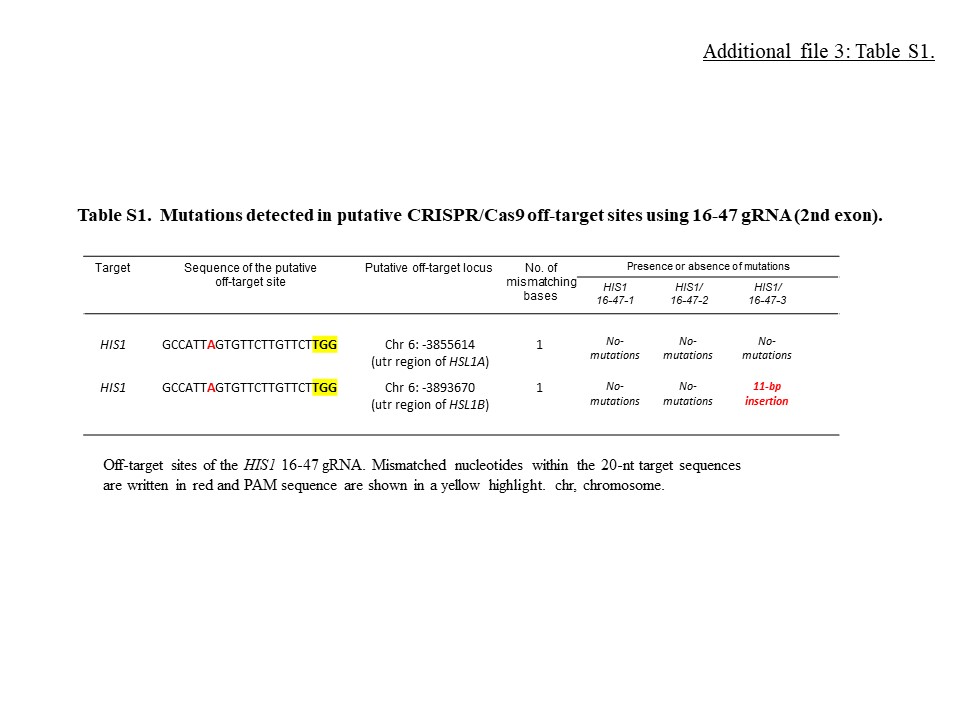

Supplement: Supplementary file 1 [file DataSheet_1.zip › Table S1..JPG]

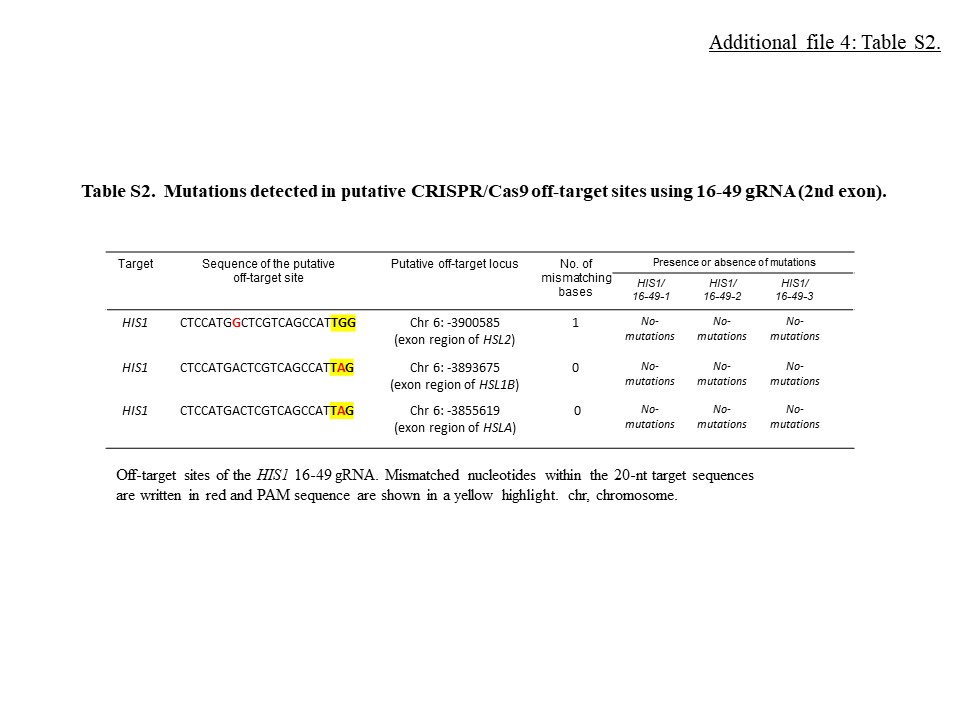

Supplement: Supplementary file 1 [file DataSheet_1.zip › Table S2..JPG]

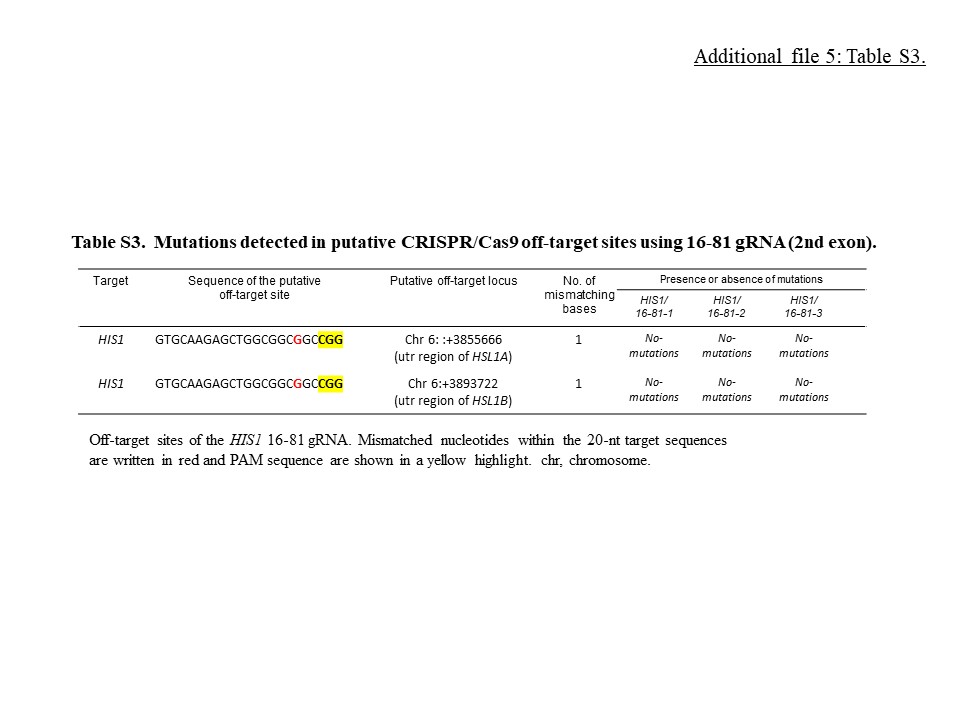

Supplement: Supplementary file 1 [file DataSheet_1.zip › Table S3.JPG]

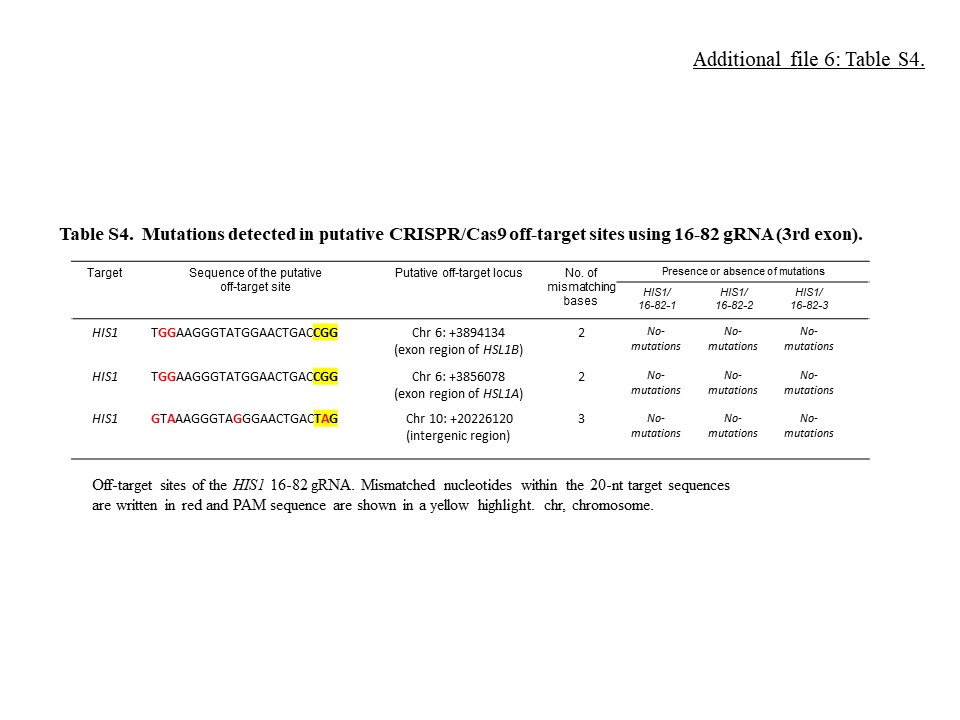

Supplement: Supplementary file 1 [file DataSheet_1.zip › Table S4..JPG]
